# Relationships among bacterial cell size, diversity, and taxonomy in rumen

**DOI:** 10.3389/fmicb.2024.1376994

**Published:** 2024-04-02

**Authors:** Sijia Liu, Nan Zheng, Jiaqi Wang, Shengguo Zhao

**Affiliations:** ^1^College of Pastoral Agriculture Science and Technology, Lanzhou University, Lanzhou, China; ^2^State Key Laboratory of Animal Nutrition and Feeding, Institute of Animal Sciences, Chinese Academy of Agricultural Sciences, Beijing, China

**Keywords:** rumen, bacteria, cell size, diversity, taxonomy

## Abstract

**Introduction:**

The rumen microbial community plays a crucial role in the digestion and metabolic processes of ruminants. Although sequencing-based studies have helped reveal the diversity and functions of bacteria in the rumen, their physiological and biochemical characteristics, as well as their dynamic regulation along the digestion process in the rumen, remain poorly understood. Addressing these gaps requires pure culture studies to demystify the intricate mechanisms at play. Bacteria exhibit morphological differentiation associated with different species. Based on the difference in size or shape of microorganisms, size fractionation by filters with various pore sizes can be used to separate them.

**Methods:**

In this study, we used polyvinylidene difluoride filters with pore sizes of 300, 120, 80, 40, 20, 8, 6, 2.1, and 0.6 μm. Bacterial suspensions were successively passed through these filters for the analysis of microbial population distribution using 16S rRNA gene sequences.

**Results:**

We found that bacteria from the different pore sizes were clustered into four branches (> 120 μm, 40–120 μm, 6–20 μm, 20–40 μm, and < 0.6 μm), indicating that size fractionation had effects on enriching specific groups but could not effectively separate dominant groups by cell size alone. The species of unclassified Flavobacterium, unclassified Chryseobacterium, unclassified *Delftia*, *Methylotenera mobilis*, unclassified Caulobacteraceae, unclassified *Oligella*, unclassified *Sphingomonas*, unclassified *Stenotrophomonas*, unclassified *Shuttleworthia*, unclassified *Sutterella*, unclassified Alphaproteobacteria, and unclassified SR1 can be efficiently enriched or separated by size fractionation.

**Discussion:**

In this study, we investigated the diversity of sorted bacteria populations in the rumen for preliminary investigations of the relationship between the size and classification of rumen bacteria that have the potential to improve our ability to isolate and culture bacteria from the rumen in the future.

## 1 Introduction

The rumen microbiota is crucial for the digestion and metabolism of feed in ruminants, with bacteria being the predominant microorganisms present (Li et al., 2019). The rumen contains a vast number of bacterial cells, estimated to be approximately 10^10^–10^11^ cells/mL, belonging to over 200 different species (Matthews et al., 2019). Rumen bacteria ferment complex carbohydrates, such as hemicellulose, cellulose, and lignin, which are inedible to humans, producing short-chain fatty acids used for dairy or meat production (Stewart et al., 2019). Maintaining homeostasis in the rumen microecology is crucial for the host’s ability to digest, feed, and survive, which ultimately affects its productivity. Therefore, manipulating rumen bacteria offers opportunities to regulate rumen metabolism and reduce food production costs.

Both cultivation and sequencing-based research have strived to reveal the diversity of rumen bacteria and their respective functionalities (Seshadri et al., 2018; Xue et al., 2020). Studies utilizing high-throughput sequencing have unveiled a remarkable abundance of bacteria in the rumen, identifying approximately 7,416 distinct microbial taxa (da Cunha et al., 2023). However, only a fraction of bacteria have been isolated from the rumen (Seshadri et al., 2018). Although sequencing-based studies have helped reveal the diversity, function, and distribution of bacteria in the rumen, their physiological and biochemical characteristics, as well as dynamic regulation along the digestion process, remain poorly understood. These aspects rely on pure culture for further elucidation.

Traditional plating cultivation methods are important for the isolation and cultivation of bacteria, which are frequently used to successfully isolate interested bacteria (Galvez et al., 2020; Sorbara et al., 2020), which is laborious and time-consuming. We need some new methods to minimize these potential limitations. Bacteria exhibit morphological differentiation associated with different species, including coccoid, rod, spirilla types, and various other uncommon shapes (Kysela et al., 2016; van Teeseling et al., 2017; Tian et al., 2023). Based on the difference in size or shape of microorganisms, size fractionation using filters with various pore sizes can be used to separate them (Sorokin et al., 2017; Lewis et al., 2020).

The distribution of bacterial species among various cell size categories in the rumen remains unreported. In this study, we investigated the diversity of sorted bacteria populations in the rumen to preliminarily explore the relationships between cell size and classification diversity, which have the potential to enhance our ability to isolate and culture bacteria from the rumen.

## 2 Methods

### 2.1 Rumen microbiota sampling

Samples of rumen fluid were obtained from three Holstein dairy cows with cannulas (weighing approximately 600 ± 50 kg) to serve as the bacterial source for rumen analysis, which is a part of our previous study and has received ethical approval (Liu et al., 2023). All the procedures involving the care and management of dairy cows were approved by the Animal Care and Use Committee for Livestock of the Institute of Animal Sciences, Chinese Academy of Agricultural Sciences (protocol no.: IAS201914). The dairy cows were fed a typical total mixed ration (DM base) consisting of 62.09% corn silage, 7.66% alfalfa hay, 10.25% corn powder, 17.87% soybean meal, and 2.13% premix. The rumen content was collected and subsequently filtered through a four-layered cheesecloth to separate the liquid. Immediately, the liquid samples were combined (in equal amounts) and injected into a penicillin bottle along with an equivalent volume of sterile glycerol (15%, v/v) using a syringe. Subsequently, they were preserved at −80°C until required.

### 2.2 Size fractionation by filters with various pore sizes

The microbiota from the rumen were fractionated based on different cell sizes using polyvinylidene difluoride filters with 300, 120, 80, 40, 20, 8, 6, 2.1, and 0.6 μm pore sizes (Merck, Merck Millipore Ltd., Ireland). The rumen fluid was successively passed through the 300, 120, 80, 40, 20, 8, 6, 2.1, and 0.6 μm filters. Four replicates were set up for each experimental treatment. To increase the flow of filtering, we used an injector, and the volumes filtered were less than 5 mL. Retentates were washed three times using a PBS solution. Bacterial fractionation resuspensions were centrifuged (13,000× *g*, 2 min, 4°C), and the sediment pellets were reserved. Consequently, bacteria fractions with size ranges at ≥300 μm, 120–300 μm (120 μm ≤ * < 300 μm), 80–120 μm (80 μm ≤ * < 120 μm), 40–80 μm (40 μm ≤ * < 80 μm), 20–40 μm (20 μm ≤ * < 40 μm), 8–20 μm (8 μm ≤ * < 20 μm), 6–8 μm (6 μm ≤ * < 8 μm), 2.1–6 μm (2.1 μm ≤ * < 6 μm), 0.6–2.1 μm (0.6 μm ≤ * < 2.1 μm), and < 0.6 μm were obtained.

### 2.3 DNA extraction and 16S rRNA gene amplification

DNA was extracted using the cetyltrimethylammonium bromide (CTAB) method plus bead beating, as previously documented (Liu et al., 2023). The quality of DNA was assessed through the utilization of agarose gel electrophoresis (1%), while the measurement of DNA concentration was conducted using a Nanodrop spectrophotometer (Thermo Scientific, United States).

The microbial diversity was detected by sequencing the V3–V4 region of 16S rRNA, which was amplified using PCR with the primers 338F (5´-ACTCCTACGGGAGGCAGCAG-3′) and 806R (5´-GGACTACHVGGGTWTCTAAT-3′). The reactions were conducted using a MyCycler Thermal Cycler (Bio-Rad, United States) following the protocol described by Liu et al. (2020). The PCR amplicons were subjected to sequencing using the Illumina MiSeq platform at Majorbio (Shanghai, China).

### 2.4 Sequencing data analysis

Sequencing raw data were analyzed using the QIIME package (Bolger et al., 2014). First, the raw reads were trimmed to remove sequencing adaptors, followed by filtering to exclude any bases containing ambiguous information. Next, paired-end reads were merged using FLASH (Magoč and Salzberg, 2011) with parameters set at an overlap of >10 bp and a mismatch rate of <20%. Chimera sequences were removed using the UCHIME *de novo* algorithm (Wang et al., 2007). Operational taxonomic units (OTUs) were grouped by applying a threshold of 0.97 similarity using the USEARCH algorithm (Caporaso et al., 2010; Edgar, 2010). Finally, the OTUs were assigned to taxa by the RDP classifier based on the Greengenes database (13_5) (Wang et al., 2007).

### 2.5 Statistics analysis

The bacterial relative abundance data were analyzed using the Kruskal–Wallis test in SAS. The difference in alpha diversity across various pore sizes was assessed using ANOSIM within the QIIME software (Liu et al., 2020). Heatmaps and heatmap diagrams were generated using MicrobiomeAnalyst (Lu et al., 2023).

### 2.6 Nucleotide sequence accession number

Data are deposited in the China National Microbiology Data Center (NMDC)^1^ with accession number NMDC10018589.^2^

## 3 Results

### 3.1 Diversity with various pore sizes

**Figure 1:**
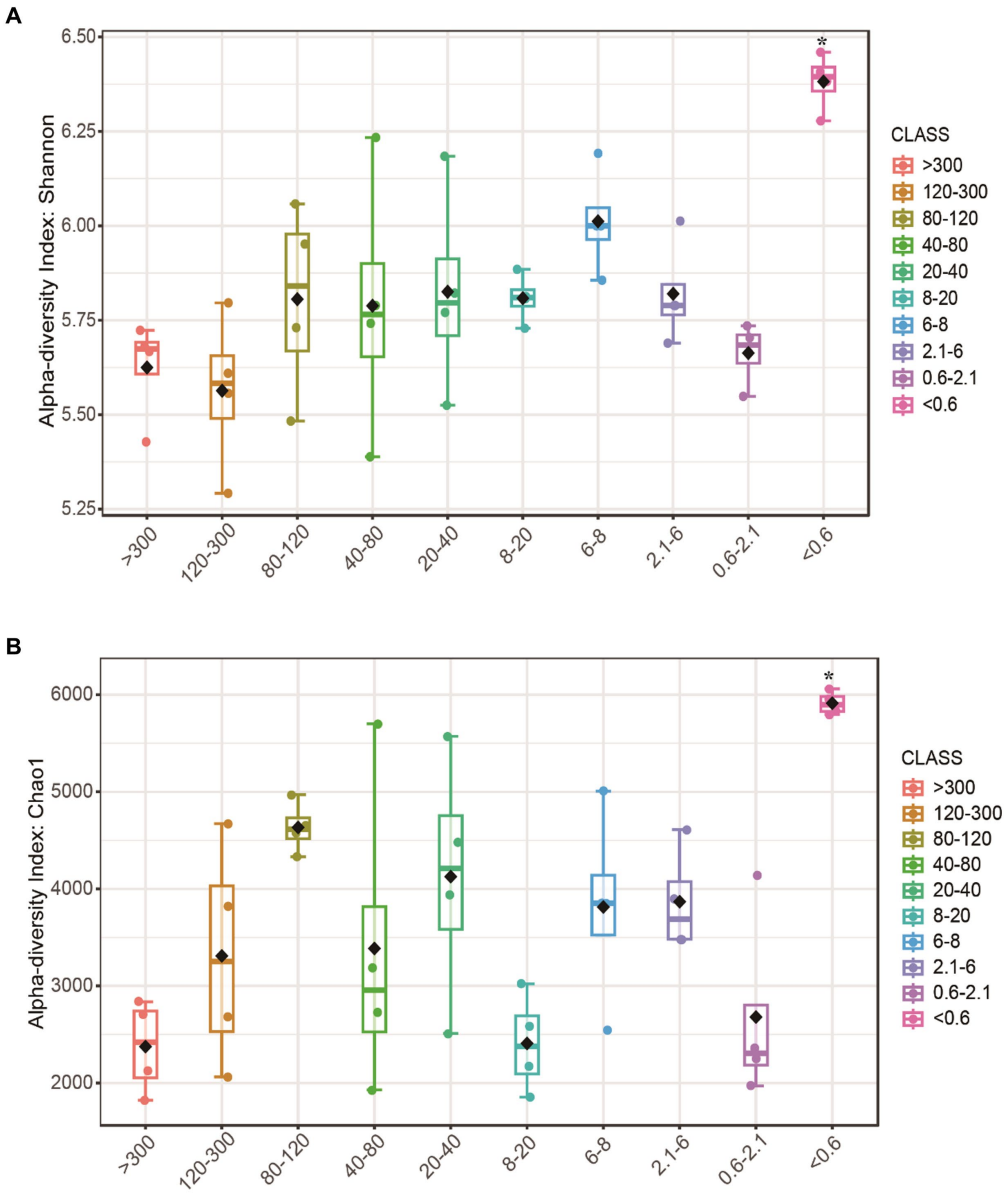
Alpha diversity of bacteria at different pore sizes. **(A)** Shannon and **(B)** Chao1 index. Boxplots indicate the first and third quartiles, with the median value indicated as a horizontal line. The whiskers extend to 1.5 times the interquartile range. Asterisk (*) indicates a significant difference.

After obtaining a total of 2,041,394 raw sequence reads, with an average of 49,510 reads, we assigned all the reads to 7,168 OTUs. The Chao1 and Shannon indices of alpha diversity in the <0.6 μm fraction were significantly higher, and there were no significant differences in the >0.6 μm fraction (Figures 1A,B).

### 3.2 Bacterial clustering with various pore sizes

**Figure 2:**
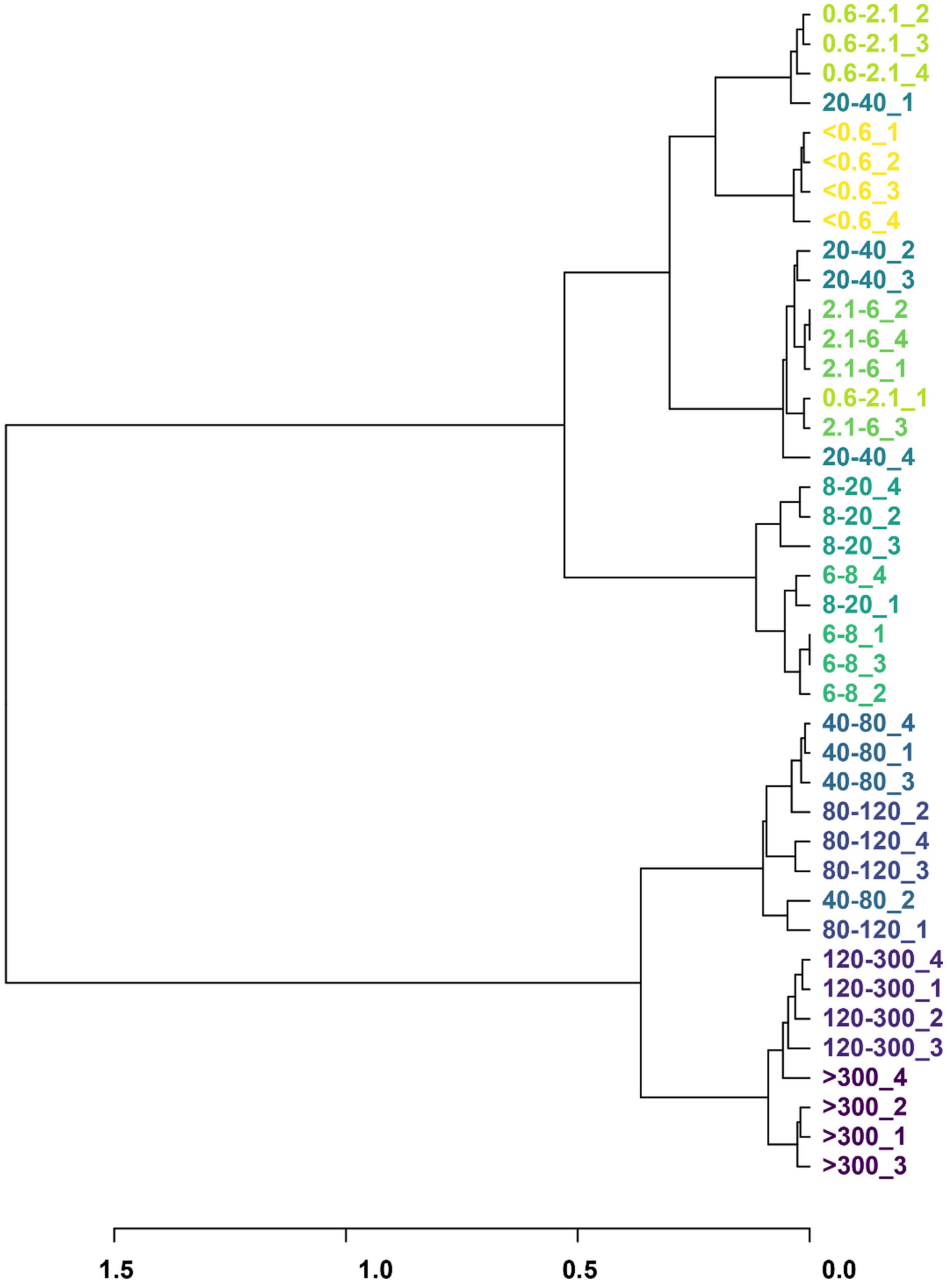
Phylogeny trees based on the bacterial classification and abundance of the cell size. Different colors represent different pore sizes, and the numbers following indicate the number of sample repetitions.

The bacteria from the various pore sizes were clustered into four branches: >120 μm, 40–120 μm, 6–20 μm, and 20–40 μm, <0.6 μm (Figure 2). The changes in community composition indicate that filtration through different cell sizes could lead to the preliminary differentiation of rumen bacteria.

### 3.3 Bacterial-specific groups with size fractionation

**Figure 3:**
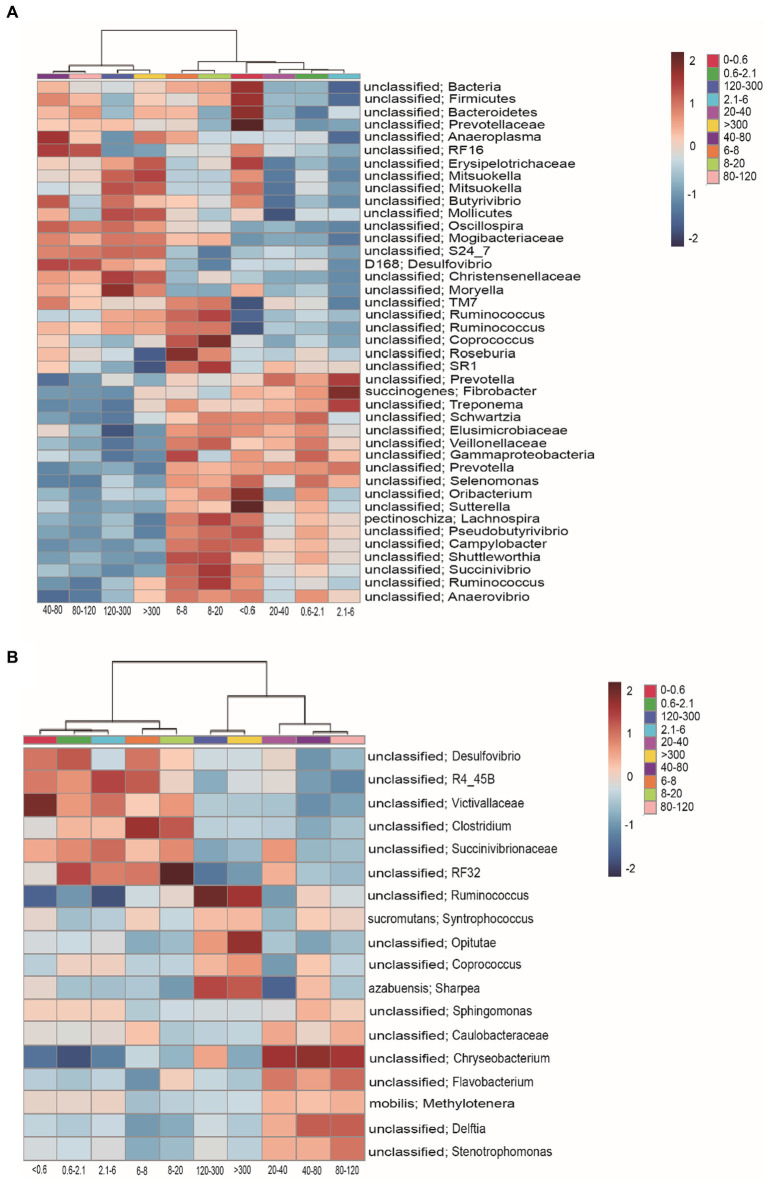
Heatmap shows the relative abundance of bacteria in the rumen of cell size. **(A)** The relative abundance of bacteria was >0.1% and **(B)** the relative abundance of bacteria was <0.1%.

The heatmap shows that the filtration method had effects on enriching specific groups of bacteria. The community of relative abundance >1% in the >40 μm fraction was unclassified Bacteria, unclassified Bacteroidetes, unclassified Firmicutes, unclassified Prevotellaceae, unclassified *Anaeroplasma*, unclassified RF16, unclassified Erysipelotrichaceae, unclassified *Mitsuokella*, unclassified *Butyrivibrio*, unclassified Mollicutes, unclassified *Oscillospira*, unclassified Mogibacteriaceae, unclassified S24_7, D168 *Desulfovibrio*, unclassified Christensenellaceae, unclassified *Moryella*, unclassified TM7, and unclassified *Ruminococcus* (Figure 3A). The 6–20 μm fraction was unclassified TM7, unclassified *Ruminococcus*, unclassified *Coprococcus*, unclassified *Roseburia*, unclassified SR1, unclassified *Schwartzia*, unclassified Elusimicrobiaceae, unclassified Veillonellaceae, unclassified Gammaproteobacteria, unclassified *Prevotella*, unclassified *Selenomonas*, unclassified Oribacterium, unclassified *Sutterella*, *Lachnospira pectinoschiza*, unclassified *Pseudobutyrivibrio*, unclassified *Campylobacter*, unclassified *Shuttleworthia*, unclassified *Succinivibrio*, and unclassified *Anaerovibrio* (Figure 3A). The <0.6 μm fraction was unclassified Bacteria, unclassified Firmicutes, unclassified Bacteroidetes, unclassified Prevotellaceae, unclassified RF16, unclassified Erysipelotrichaceae, unclassified *Mitsuokella*, *Fibrobacter succinogenes*, unclassified *Treponema*, unclassified *Schwartzia*, unclassified Elusimicrobiaceae, unclassified Veillonellaceae, unclassified Gammaproteobacteria, unclassified *Prevotella*, unclassified *Selenomona*, unclassified Oribacterium, unclassified *Sutterella*, *Lachnospira pectinoschiza*, unclassified *Pseudobutyrivibrio*, unclassified *Campylobacter*, unclassified *Shuttleworthia*, unclassified *Succinivibrio*, and unclassified *Anaerovibrio* (Figure 3A). The 2.1–6 μm fraction was unclassified *Prevotella*, *Fibrobacter succinogenes*, and unclassified *Treponema* (Figure 3A).

The community of relative abundance <1% in the <20 μm fraction was unclassified *Desulfovibrio*, unclassified R4_45B, unclassified Victivallaceae, unclassified Clostridium, unclassified Succinivibrionaceae, and unclassified RF32 (Figure 3B). The 20–120 μm fraction was unclassified *Sphingomonas*, unclassified Caulobacteraceae, unclassified Chryseobacterium, unclassified Flavobacterium, *Methylotenera mobilis*, unclassified *Delftia*, and unclassified *Stenotrophomonas* (Figure 3B). The >120 μm fraction was unclassified *Ruminococcus*, *Syntrophococcus sucromutans*, unclassified Opitutae, unclassified *Coprococcus*, and *Sharpea azabuensis* (Figure 3B).

### 3.4 Enrichment-specific bacteria with size fractionation

**Figure 4:**
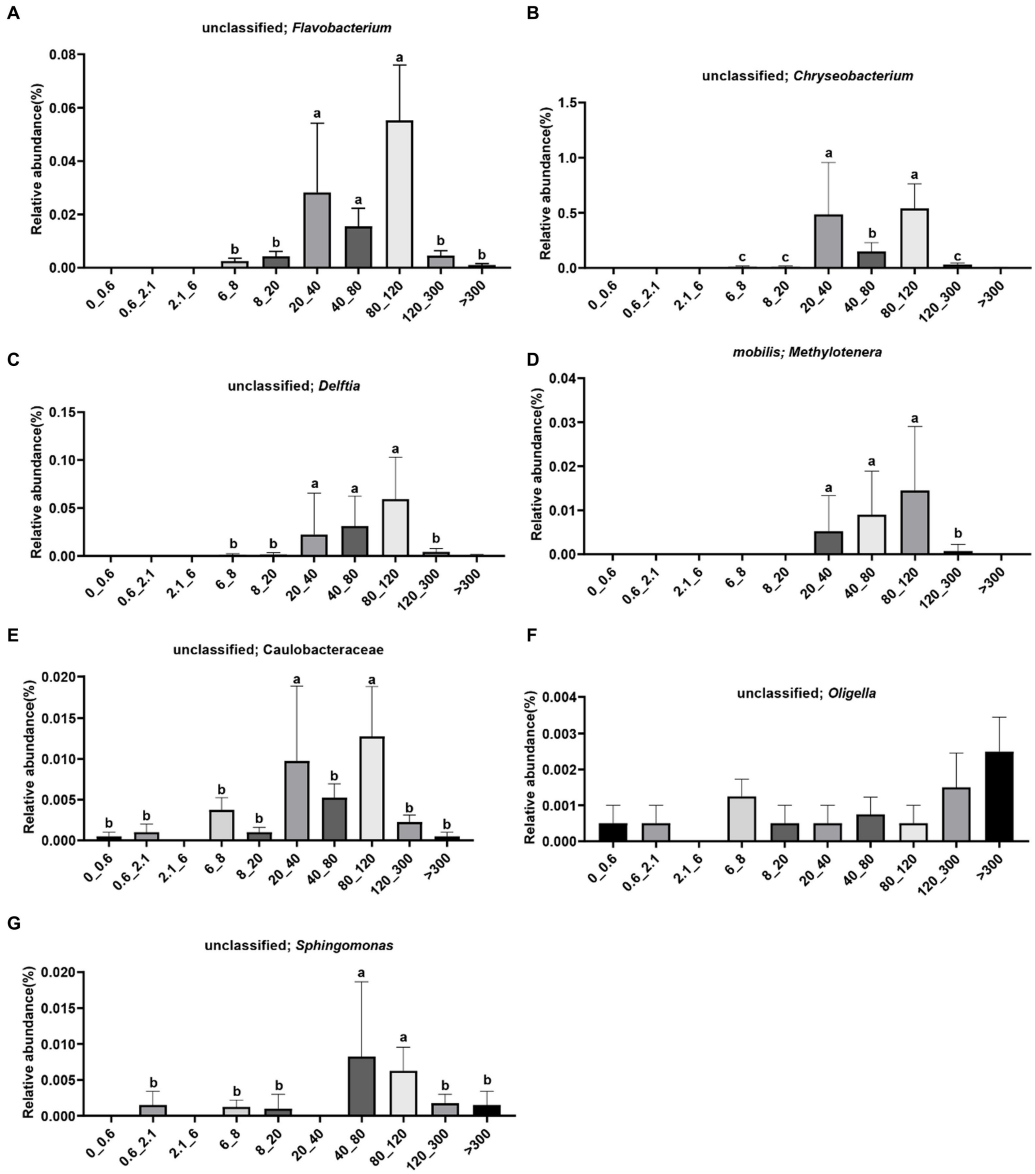
Distribution of bacteria in different pore sizes. **(A)** Unclassified; *Flavobacterium*, **(B)** unclassified; *Chryseobacterium*, **(C)** unclassified; *Delftia*, **(D)** mobilis; *Methylotenera*, **(E)** unclassified; *Caulobacteraceae*, **(F)** unclassified; *Oligella*, **(G)** unclassified; *Sphingomonas*. The letters on the pillars indicate a significant difference.

The species of unclassified Flavobacterium were enriched in the >6 μm fraction, with significantly higher abundance observed in the 20–120 μm fraction (Figure 4A). The species of unclassified Chryseobacterium were enriched in the 6–300 μm fraction, with significantly higher abundance observed in the 20–40 μm and 80–120 μm fractions (Figure 4B). The species of unclassified *Delftia* were enriched in the >6 μm fraction, with significantly higher abundance observed in the 20–120 μm fraction (Figure 4C). The species of *Methylotenera mobilis* were enriched in the 20–300 μm fraction, with significantly higher abundance observed in the 20–120 μm fraction (Figure 4D). The species of unclassified Caulobacteraceae were not enriched in the 2.1–6 μm fraction, with significantly higher abundance observed in the 20–40 μm and 80–120 μm fractions (Figure 4E). The species of unclassified *Oligella* were not enriched in the 2.1–6 μm fraction, with higher abundance observed in the >300 μm fraction (Figure 4F). The species of unclassified *Sphingomonas* were not enriched in the <0.6 μm, 2.1–6 μm, and 20–40 μm fractions, with significantly higher abundance observed in the 40–120 μm fraction (Figure 4G).

**Figure 5:**
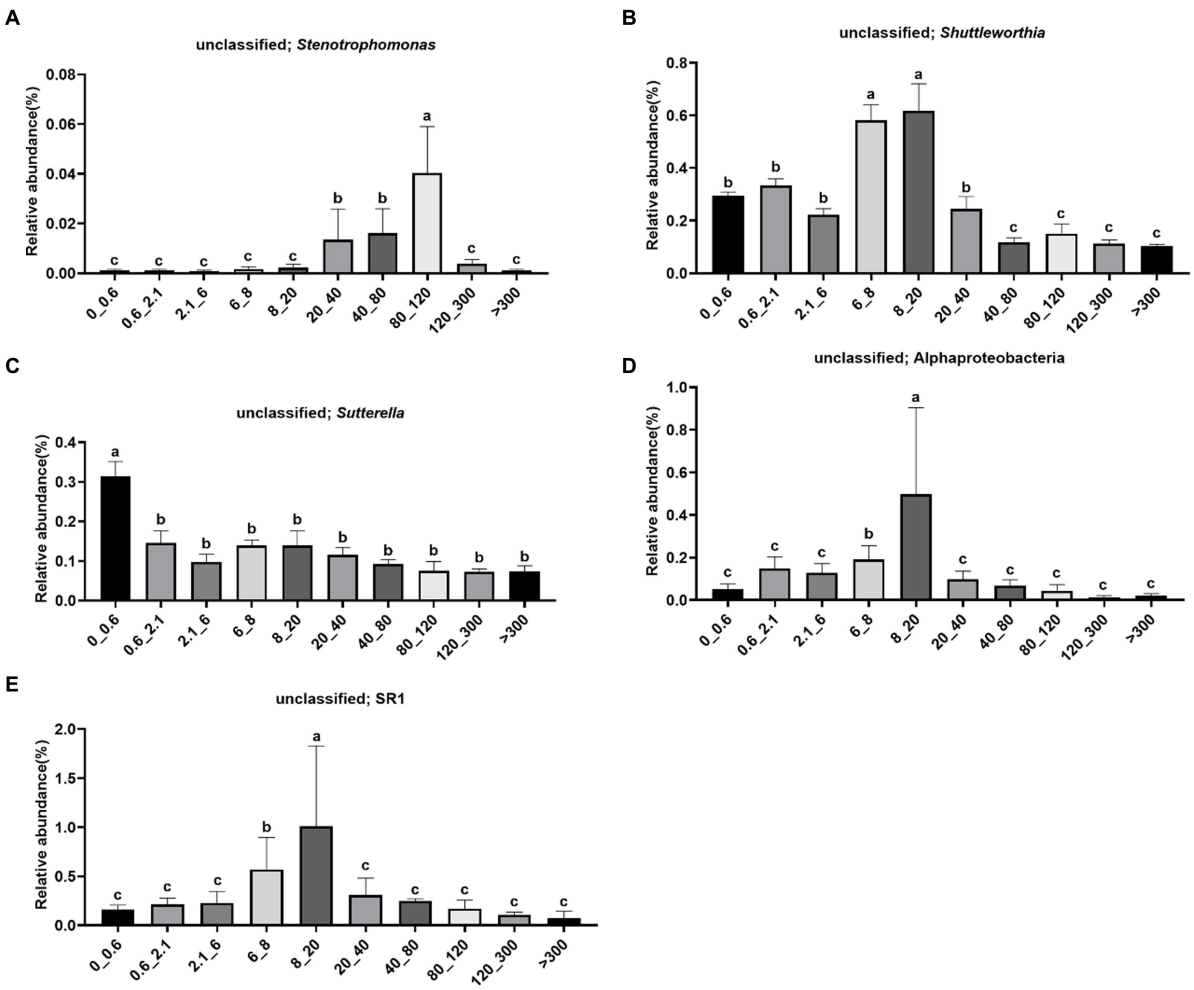
Relative abundance of bacteria in different pore sizes. **(A)** Unclassified; *Stenotrophomonas*, **(B)** unclassified; *Shuttleworthia*, **(C)** unclassified; *Sutterella*, **(D)** unclassified; Alphaproteobacteria, **(E)** unclassified; SR1. The letters on the pillars indicate a significant difference.

The abundance of unclassified *Stenotrophomonas* was significantly higher in the 80–120 μm fraction (Figure 5A). The abundance of unclassified *Shuttleworthia* was significantly higher in the 6–20 μm fraction (Figure 5B). The abundance of unclassified *Sutterella* was significantly higher in the <0.6 μm fraction (Figure 5C). The abundance of unclassified *Alphaproteobacteria* was significantly higher in the 8–20 μm fraction (Figure 5D). The abundance of unclassified SR1 was significantly higher in the 8–20 μm fraction (Figure 5E).

## 4 Discussion

Ruminal bacterial communities are vast and complex, with only a subset of the species participating in specific functions (Xue et al., 2020; Sun et al., 2021). Therefore, the primary question is to determine which members of the ruminal bacteria community are responsible for specific functions of interest, with the goal of isolating and studying them to regulate their metabolism and improve ruminal production efficiency (Pope et al., 2011). However, effective methods for enriching and isolating rumen microorganisms are currently lacking. In this study, we successfully revealed the relationship between the size and classification of rumen bacteria. The application of this method to enrich interested bacterial groups may provide an opportunity to capture key species in specific metabolic pathways.

Different approaches have been used to decrease the bacterial diversity in complex communities prior to cultivation, including techniques such as filtration, density-gradient centrifugation or elutriation, and serial dilution-to-extinction (Bernard et al., 2000; Vartoukian et al., 2010). Filtration has been widely utilized in numerous studies, establishing itself as a prevalent technique with proven efficacy (Sorokin et al., 2017; Hu et al., 2021). In this study, we used a sequential filtration method that utilized different pore sizes to achieve the separation of different size fractions within the complex ruminal community. Through this method, rumen bacteria were divided into four clusters corresponding to different cell sizes, and the diversity was reduced, which indicates filtration is more suitable for further enrichment before bacteria isolation and cultivation.

Some studies revealed that *Prevotella*, *Butyrivibrio*, and *Ruminococcus* were the dominant bacterial groups in the rumen using sequencing technology (Henderson et al., 2015; Matthews et al., 2019). As the largest number of bacteria in the rumen, they have great genetic differences among species, which is closely related to healthy rumen metabolism (La Reau et al., 2016; Palevich et al., 2019; Dao et al., 2021; Betancur-Murillo et al., 2022). However, these dominant groups cannot be effectively separated from each other by cell size. One potential explanation is that cell aggregates may form either autonomously or through alternative mechanisms of adhesion to diverse cellular entities (Kubo et al., 2012; Garrison and Bochdansky, 2015). Only some rare and unclassified species can be efficiently enriched or separated by cell size. Most of the groups we identified were unclassified, potentially due to limitations in accurately assigning taxonomy using commonly targeted 16S sub-regions compared to sequencing the full 16S gene or metagenome (Johnson et al., 2019). With the continuous development of sequencing technology and the reduction in costs, the use of metagenomic sequencing or third-generation sequencing technology in the future may be able to address this issue.

Taken together, our results illustrate that although the most interesting bacteria cannot be targeted based on cell size in the rumen, we can enrich the targeted group through filtration. Additionally, when combined with a specific method, such as Raman-activated cell sorting (Lee et al., 2019) and live-FISH (Batani et al., 2019), or a high-throughput method, for example, culturomics (Mailhe et al., 2018) and iChip (Nichols et al., 2010), we can effectively isolate bacteria from complex communities and environments.

## 5 Conclusion

In this study, we investigated the relationship between the size and classification of rumen bacteria. The results showed that the dominant bacterial groups could not be effectively separated from each other based on cell size alone. A small proportion of rare unclassified bacterial groups could be efficiently enriched or separated by cell size, which may enhance our ability to isolate, culture, and characterize specific microorganisms from the rumen in future studies.

## Data availability statement

The datasets presented in this study can be found in online repositories. The names of the repository/repositories and accession number(s) can be found at: https://nmdc.cn/resource/genomics/project/detail/NMDC10018589, NMDC10018589.

## Author contributions

SL: Data curation, Formal analysis, Investigation, Methodology, Writing – original draft. NZ: Resources, Writing – review & editing. JW: Funding acquisition, Supervision, Writing – review & editing. SZ: Investigation, Methodology, Resources, Writing – review & editing.

## Footnotes

^1^ https://nmdc.cn/en

^2^ https://nmdc.cn/resource/genomics/project/detail/NMDC10018589
